# Exercise and dietary change ameliorate high fat diet induced obesity and insulin resistance via mTOR signaling pathway

**DOI:** 10.20463/jenb.2016.06.20.2.4

**Published:** 2016-06-30

**Authors:** Ju Yong Bae, Ki Ok Shin, Jinhee Woo, Sang Heon Woo, Ki Soeng Jang, Yul Hyo Lee, Sunghwun Kang

**Affiliations:** 1 Laboratory of Exercise Biochemistry, Department of Physical Education, Dong-A University, Busan Republic of Korea; 2 Laboratory of Exercise physiology, Division of Sport Science, Kangwon National University, Chuncheon Republic of Korea

**Keywords:** Training, Aerobic exercise, Regular exercise, Insulin receptor substrate, mammalian target of rapamycin, Akt

## Abstract

**[Purpose]:**

The purpose of this study was to investigate the effect of exercise and dietary change on obesity and insulin resistance and mTOR signaling protein levels in skeletal muscles of obese rats.

**[Methods]:**

Sixty male Sprague-Dawley rats were divided into CO (Normal diet) and HF (High Fat diet) groups in order to induce obesity for 15 weeks. The rats were then subdivided into CO, COT (CO + Training), HF, HFT (HF + Training), HFND (Dietary change), and HFNDT (HFND + Training) groups (10 rats / group). The training groups underwent moderate-intensity treadmill exercise for 8 weeks, after which soleus muscles were excised and analyzed. Data was statistically analyzed by independent t-test and One-way ANOVA tests with a 0.05 significance level.

**[Results]:**

Fasting blood glucose, plasma insulin, and HOMA-IR in the HF group were significantly higher, as compared with other groups (p <.05). Protein levels of insulin receptor subunit-1 (IRS-1), IRS-2, and p-Akt were significantly higher in the HFT, HFND, and HFNDT groups, as compared with HF group. In addition, the protein levels of the mammalian target of rapamycin complex 1 (mTORC1) and ribosomal S6 protein kinase 1 were significantly decreased by exercise and dietary change (p <.05). However, mTORC2 and phosphoinositide 3-kinase were significantly increased (p <.05).

**[Conclusion]:**

In summary, despite the negative impact of continuous high fat intake, regular exercise and dietary change showed a positive effect on insulin resistance and mTOR signaling protein levels.

## INTRODUCTION

Increased occurrence of lipid species such as triglyceride, diacylglycerol, and ceramide in skeletal muscle, reportedly increases stored fat mass on consumption of high fat diet, resulting in impairment of insulin signaling pathway and glucose transfer system^1^. Over-intake of a high fat diet leads to metabolic syndrome, and excessive eating of carbohydrates and proteins also affects insulin resistance^2,3^. Insulin resistance induced by a high fat diet involves decreased protein levels of insulin signaling pathway factors, such as insulin receptor (IR), phosphoinositide 3-kinase (PI3K), and Akt^4,5^.

Dietary overindulgence activates mTORC1 that is stimulated by mTOR, inhibits the activity of PI3K through phosphorylation of ribosomal S6 protein kinase 1 (S6K1) to decompose amino acid^6^, and results in preventing phosphorylation of tyrosine and suppression of insulin receptor substrate 1 (IRS-1)^7-9^. Inhibition of IRS-1 through reduction of tyrosine phosphorylation and S6K1 phosphorylation also results in the activation of mTORC1^10,11^. Therefore, mTORC1 activation in the skeletal muscle could induce insulin resistance. However, the role of mTORC2 associated with insulin signaling is currently unknown.

Endurance training and regular exercise have positive effects on insulin action and mTOR signaling. A serine-threonine protein kinase (AKT) is activated by PI3K pathway through exercise in the form of external stimulation of the cell^12^, and regulates cellular organization and hypertrophy^13,14^. However, the effect of exercise and/or dietary change on the metabolic pathways associated with continuous high fat diet-induced obesity is currently unclear. In addition, the precise mechanisms by which exercise and/or dietary change affect upstream and downstream mTOR signaling pathway related in insulin resistance is currently unknown.

Therefore, the objective of this study was to investigate the effect of exercise and dietary change on obesity and insulin resistance and mTOR signaling protein levels in skeletal muscle of obese rats induced by high fat diet for 15 weeks.

## METHODS

### Experimental animals and treatments

Sixty male Sprague Dawley (SD, 4 weeks old) rats were housed in cages and fed freely with standard rat chow and water (Daehan Biolink, Korea). Three or 4 rats were housed in each cage, and maintained under standardized conditions in an animal facility (Laboratory of animals, College of Medicine, Dong-A University), with a room temperature of 22 ± 1.5°C, 50 ~ 60 % relative humidity, and a 12 hour light/dark cycle. All rats were cared for during the entire period of experimentation in accordance with the Guidelines of Animal Experiments recommended by the Institutional Animal Care and Use Committee. After 1 week of adaptation maintenance, rats were randomly divided into two groups to induce obesity by high fat diet for 15 weeks: a normal diet (CO) group (12 % fat; Donga SF, Korea, n = 20), and a high fat diet (HF) group (40 % fat; AIN-76A; Jungang Lab Animal, Inc, Korea, n = 40). Body weight was measured every week during the entire experimental period.

### Exercise program

After inducing obesity, rats were randomly subdivided into the CO, COT (CO + training), HF, HFT (HF + training), HFND (dietary change to normal diet), and HFNDT (HFND + training) groups (10 rats per group). Rats in the exercise training groups were put on a treadmill for 40 min once a day, 5 times a week, for 8 weeks. Exercise intensity consisted of 5 m/min (5 min), 12 m/min (5 min), and 18m/min (20 min) at 0% slope for weeks 1 to 4 (low intensity). During weeks 5 to 8, exercise intensity was increased to 10 m/min (5 min), 16 m/min (5 min), and 22 m/min (30 min) at the same slope (moderate intensity)^15^.

### Blood and tissue samplings

To exclude the temporary effects of treadmill exercise, sacrifice was conducted after 48 hours from the last exercise session. After complete anesthesia (ethyl ether), blood samples (5 ml) from the abdominal vena cava were obtained in syringes. Plasma was collected by centrifugation of heparinized blood at 3000 rpm for 15 min. Soleus muscle were removed and stored at -70°C until analysis.

### Lipid profiles

Plasma total cholesterol (TC) and triglyceride (TG) levels were analyzed with rat TC and TG kits (Asan Pharmaceutical, Korea). High density lipoprotein cholesterol (HDL-c) level was analyzed with HDL-c kits (Shinyang Diagnostics, Korea) and Low density lipoprotein cholesterol (LDL-c) was calculated with the following equation: LDL-c = TC - (HDL-c + TG/5)16. Plasma insulin level was analyzed with a rat insulin ELISA kit (Shibayagi Co. Ltd, Japan) according to the manufacturer’s instructions. Blood glucose level was estimated using a GlucoDr glucometer (Allmedicus, Korea). Insulin resistance index (IRI) was assessed by homeostasis model assessment estimate of insulin resistance (HOMA-IR) as follows:

IRI = Fasting insulin (µIU/mL) X Fasting glucose (mg/dL) / 405

### Western blot

To extract protein from the soleus muscle, tissues were homogenized after adding a solution containing 150 mM NaCl, 5 mM EDTA, 50 mM Tri-HCl (pH 8.0), 1 %-NP 40, 1 mM aprotinin, 0.1 mM leupeptin, and 1 mM pepstatin. The solution was centrifuged for 30 minutes at 13,000 rpm. Supernatants were collected and assayed for protein content prior to storage at -70°C. Protein samples were mixed with Laemmli sample buffer (LSB) and placed in a boiling water bath for 5 min. Proteins were resolved by 10, 12 or 15 % SDS-polyacrylamide gel electrophoresis (SDS-PAGE; each loaded with same μg of total protein per lane), and transferred to nitrocellulose membranes. Proteins on the membranes were blocked in 5 % skim milk in phosphate – buffered saline (PBS) (NaCl 8 g, KCl 0.2 g, Na_2_HPO_4_ 1.44 g, KH_2_PO_4_ 0.24 g, pH 7.4). Thereafter, protein membranes were incubated with the following primary antibodies: IRS-1, 2 (#2382, #4502, Cell Signaling), Akt (#9272, Cell Signaling), p-Akt (#4060, Cell Signaling), mTOR (#2972, Cell Signaling), mTORC1 (#2280, Cell Signaling), mTORC2 (#2114, Cell Signaling), PI3K (#4255, Cell Signaling), S6K1 (#9202, Cell Signaling) for one hour, and washed thrice (15 min each) in a PBS solution containing 0.1 % tween 20. Washed membrane was then treated with secondary antibody (goat anti mouse or rabbit IgG) conjugated with horseradish peroxidase (HRP). Immune-reactive bands were developed on Kodak film. The relative strengths of bands were quantitated by densitometry (Sci – Scan, UUSB).

### Data analysis

The change in body weight induced by high fat diet was analyzed by independent t-test and ANOVA. One-way ANOVA and Duncan’s post-hoc analysis were performed for any intergroup difference observed. All data were tested for normal distribution using the Shapiro-Wilk test. All data were analyzed using SPSS Software Version 21.0 for Windows (SPSS Inc. Chicago. IL). Data were expressed as mean ± standard error (SE). Statistical significance was defined as a p value < 0.05.

## RESULTS

**Figure 1.**
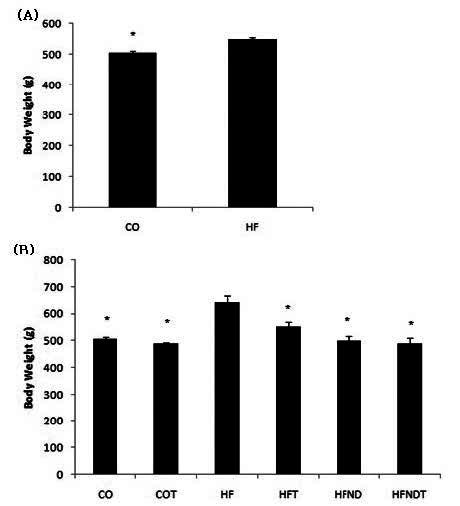
mean±SE, (A) Changes of body weight after 15 weeks of high-fat diet, (B) Changes of body weight after exercise and dietary change for 8 weeks. ^*^p<0.05 vs HF. CO; normal diet group, COT; normal diet + training group, HF; high fat diet group, HFT; high fat diet + training group, HFND; dietary change group, HFNDT; dietary change + training group.

After 15 weeks of a high fat diet, body weight in HF group was significantly higher, as compared to the CO group (p <.05) (Figure 1-A). After 8 weeks of exercise and dietary change, body weight in the HFT, HFND, and HFNDT groups was significantly lower than in the HF group (p <.05) (Fig. 1-B).

TC was significantly higher in the HF group than in the COT, HFT, HFND, and HFNDT groups (p <.05). TG was higher in the HF group, as compared with all other groups (p <.05). HDL-c level was significantly lower in the HF group than in the COT and HFNDT groups (p <.05). LDL-c level was significantly higher in the HF group than in the HFNDT group (p <.05). Glucose level was significantly higher in the HF group than in the CO, COT, and HFNDT groups (p <.05). Insulin and HOMA-IR levels were significantly higher in the HF group, as compared with all other groups (p <.05) (Table 1).

**Table 1.** Change of lipid profiles after 8 weeks treatment

variable	CO	COT	HF	HFT	HFND	HFNDT
TC(mg/dl)	191.55	167.01*	201.35	163.80*	167.32*	165.05*
	±2.85	±5.91	±11.16	±15.92	±5.23	±7.81
TG(mg/dl)	76.25*	73.16*	115.52	68.09*	74.37*	80.36*
	±3.89	±4.57	±14.20	±7.82	±8.67	±11.58
HDL-c(mg/dl)	33.37	36.84*	26.93	33.26	29.95	35.81*
	±1.63	±2.30	±2.05	±2.54	±1.92	±2.21
LDL-c(mg/dl)	142.93	115.54	151.42	116.93	122.50	113.17*
	±3.43	±6.58	±11.24	±18.02	±5.65	±8.78
Glucose(mg/dl)	139.00*	135.38*	170.00	147.27	149.08	138.75*
	±3.09	±2.34	±10.80	±6.43	±8.55	±3.62
Insulin(uIU/ml)	31.04*	18.93*	69.97	40.42*	39.88*	24.03*
	±6.38	±2.65	±14.57	±8.30	±9.10	±3.75
HOMA-IR	10.70*	6.31*	28.39	14.66*	14.80*	8.26*
	±2.30	±0.85	±5.12	±2.95	±3.47	±1.34

mean±SE, *vs HF p<0.05. TC; total cholesterol, TG; triglycerides, HDL-c; high density lipoprotein cholesterol, LDL-c; low density lipoprotein cholesterol, HOMA-IR; homeostatic model assessment insulin resistance.

**Figure 2.**
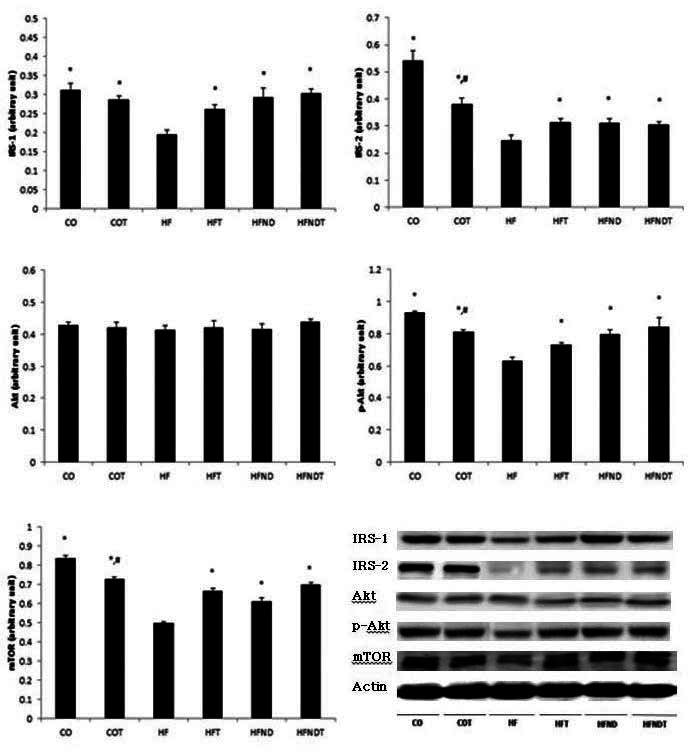
mean±SE, ^*^p<0.05 vs HF, ^#^p<0.05 vs CO. CO; normal diet group, COT; normal diet + training group, HF; high fat diet group, HFT; high fat diet + training group, HFND; dietary change group, HFNDT; dietary change + training group.

After 8 weeks of exercise and dietary change, muscle protein level of IRS-1, IRS-2, p-Akt, and mTOR in the HF group were significantly lower, as compared with all other groups (p <.05). Also, muscle protein level of IRS-2, p-Akt and mTOR in the CO group were significantly higher, as compared with the COT group (p <.05). However, Akt protein level was not significantly different, as compared with all other groups (Fig. 2).

**Figure 3.**
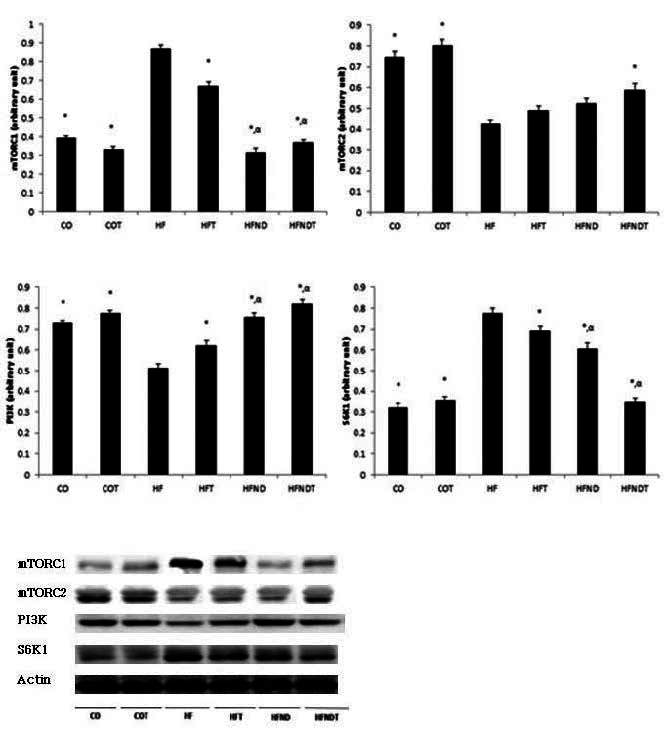
mean±SE, ^*^p<0.05 vs HF, αp<0.05 vs HFT. CO; normal diet group, COT; normal diet + training group, HF; high fat diet group, HFT; high fat diet + training group, HFND; dietary change group, HFNDT; dietary change + training group.

The mTORC1 protein level was significantly higher in the HF group than in all other groups (p <.05). In addition, mTORC1 protein level were significantly lower in the HFND and HFNDT groups, as compared with the HFT group (p <.05). mTORC2 protein levels of were significantly higher in the CO, COT, and HFNDT groups, as compared with the HF group (p <.05). The PI3K protein level was significantly lower in the HF group, as compared with all other groups (p <.05). In addition, the HFND and HFNDT groups showed significantly higher levels of PI3K compared with the HFT group (p <.05). S6K1 protein level was significantly higher in the HF group, as compared with all other groups (p <.05). In addition, the HFND and HFNDT groups showed significantly lower levels of S6K1, as compared with the HFT group (p <.05) (Fig. 3).

## DISCUSSION

Regular exercise and dietary change can be effective for regulation of body weight and insulin resistance. Previous studies have reported that regular exercise has positive effects on insulin resistance in skeletal muscle via mTOR signaling pathway. We aimed to determine the effect of exercise and dietary change on biochemical changes of mTOR signaling pathway, in case of obesity induced by continuous consumption of high fat diet. Therefore, we evaluated the insulin resistance and mTOR signaling protein levels in skeletal muscle of high fat diet induced obese rats after 8 weeks of regular exercise and dietary change.

Excessive intake of carbohydrates, proteins, and fats negatively affects insulin action in skeletal muscle^17,18^, partly via glucose metabolism^1^. High fat diet induced obesity reportedly causes insulin resistance and leptin resistance in peripheral tissues^19,20^. A member of the mTOR signaling pathway, mTORC1, is known to negatively control insulin levels through inhibition of IRS-19. Although mTORC2 prevents the activation of S6K121, the effect of mTORC2 on insulin activity is currently unclear. However, mTORC2 controls the activation and phosphorylation of Akt, and reduced mTORC2 in skeletal muscle leads to decreased glucose uptake via insulin induction^22^. Therefore, mTORC2 and Akt may play important roles in the activation of insulin that is essential for glucose homeostasis. In this study, we confirmed that protein levels of mTORC2, PI3K, and p-Akt were significantly decreased by high fat diet. In addition, high fat diet induced obese rats had high protein levels of mTORC1 and S6K1, but low protein levels of IRS-1 and IRS-2. Therefore, high fat diet-induced metabolic pathway showed a negative effect on insulin action in skeletal muscle.

Exercise increases metabolism in skeletal muscle by improving insulin action and glucose uptake^23,24^, is suggested as the most effective method for the treatment of insulin resistance in skeletal muscle^25^. A previous study reported that exercise has a positive effect on insulin resistance^19^ and leptin resistance^20^ that is mediated by improvements in glucose metabolism. Exercise-induced mTOR complex activity suggests that phosphatidic acid is stabilized by the formation of mTOR complexes, therefore regulating mTORC1 in response to metabolic stimulation by nutrients and growth factors^26,27^. A previous study reported that acute exercise increases activation of mTOR for a few hours^28^. In addition, both aerobic exercise through the various muscle contractions^29^ and high-intensity resistance exercise^30^ activate mTOR. Previously, a report indicated that regular exercise regulates the insulin signaling pathway via PI3K activation^31^. Chibalin et al., demonstrated that swimming exercise changes the level of IR in Wistar rats^32^; however, this is unlikely to be the sole explanation for the increased metabolic response to insulin, because insulin stimulates glucose transport activity, as evidenced by a dramatic increase in GLUT4 protein expression after just one day of exercise. Another study showed that 6 weeks exercise improved skeletal muscle insulin resistance without reduced mTOR/S6K1 signaling pathway^33^. However, the body weight of rats showed no increase in response to 59% high-fat diet for 6 weeks, and exercise for 6 weeks^33^. The discrepancy in results of previous study as compared to the present study, is possibly due to insufficient exercise duration, and differences in fat composition.

Dietary intervention is another effective method for the treatment of insulin resistance. Many studies report that caloric restriction increases insulin sensitivity^34,35^, and this result might be due to the reduced body weight and fat mass^36^. In addition, previous studies conducted a combination of exercise and/or dietary restriction to analyze changes in glucose metabolism^37,38^. Ross et al., reported physical activity without caloric restriction increased weight loss, and substantially reduces insulin resistance^37^; and Larson-Meyer et al., reported caloric restriction alone or with exercise ameliorated insulin resistance^36^. In this study, we confirmed that regular exercise and/or dietary change reduces the level of mTORC1 but activates mTORC2, suggesting that mTORC1 and mTORC2 may act to ameliorate obesity and insulin resistance.

In the present study, we show a negative effect of the mTOR signaling pathway in obese rats induced by high fat diet. However, regular exercise and dietary change directly brought about improvements of glucose metabolism and insulin level. In addition, our study demonstrated that regular exercise decreases mTORC1 levels, and the exercise, dietary change, and combination of treatments ameliorate obesity and insulin resistance in skeletal muscle via increasing mTORC2 and p-Akt protein levels.

In summary, regular exercise and/or dietary change ameliorates obesity and insulin resistance via regulating mTORC1 and mTORC2 protein. Therefore, despite the negative impact of continuous high fat diet intake, regular exercise and dietary change results in a positive effect on insulin resistance and mTOR signaling protein levels.

## References

[1] Hawley JA., Lessard SJ. (2008). Exercise training-induced improvements in insulin action. *Acta Physiol (Oxf)*.

[2] Kuate D., Kengne AP., Biapa CP., Azantsa BG. (2015). Abdul Manan Bin Wan Muda W. Tetrapleura tetraptera spice attenuates high-carbohydrate, high-fat diet-induced obese and type 2 diabetic rats with metabolic syndrome features. *Lipids Health Dis*.

[3] Um SH., D’Alessio D., Thomas G. (2006). Nutrient overload, insulin resistance, and ribosomal protein S6 kinase 1, S6K1. *Cell Metab*.

[4] Björnholm M., Zierath JR. (2005). Insulin signal transduction in human skeletal muscle: identifying the defects in Type II diabetes. *Biochem Soc Trans*.

[5] Lessard SJ., Rivas DA., Chen ZP., Bonen A., Febbraio MA., Reeder DW., Kemp BE., Yaspelkis BB., Hawley JA. (2007). Tissue-specific effects of rosiglitazone and exercise in the treatment of lipid-induced insulin resistance. *Diabetes*.

[6] Dennis PB., Jaeschke A., Saitoh M., Fowler B., Kozma SC., Thomas G. (2001). Thomas G. Mammalian TOR: a homeostatic ATP sensor. *Science*.

[7] Fang Y., Vilella-Bach M., Bachmann R., Flanigan A., Chen J. (2001). Phosphatidic acid-mediated mitogenic activation of mTOR signaling. *Science*.

[8] Patti ME., Brambilla E., Luzi L., Landaker EJ., Kahn RC. (1998). Bidirectional modulation of insulin action by amino acids. *J Clin Invest*.

[9] Sun Y., Fang Y., Yoon MS., Zhang C., Roccio M., Zwartkruis FJ. (2008). Phospholipase D1 is and effector of Rheb in the mTOR pathway. *Proc Natl Acad Sci U S A*.

[10] Tremblay F., Marette A. (2001). Amino acid and insulin signaling via the mTOR/p70 S6kinase pathway. A negative feedback mechanism leading to insulin resistance in skeletal muscle cells. *J Biol Chem*.

[11] Tremblay F., Brûlé S., Hee Um S., Li Y., Masuda K., Roden M., Sun XJ., Krebs M., Polakiewicz RD., Thomas G., Marette A. (2007). Identification of IRS-1 Ser-1101 as a target of S6K1 in nutrient- and obesity-induced insulin resistance. *Proc Natl Acad Sci U S A*.

[12] Datta SR., Brunet A., Greenberg ME. (1999). Cellular survival: a play in three Akts. *Genes Dev*.

[13] Chen WS., Xu PZ., Gottlob K., Chen ML., Sokol K., Shiyanova T., ninson I., Weng W., Suzuki R., Tobe K., Kadowaki T., Hay N. (2001). Growth retardation and increased apoptosis in mice with homozygous disruption of the Akt1 gene. *Genes Dev*.

[14] Verdu J., Buratovich MA., Wilder EL., Birnbaum MJ. (1999). Cell-autonomous regulation of cell and organ growth in Drosophila by Akt/PKB. *Nat Cell Biol*.

[15] Woo J., Shin KO., Park SY., Jang KS., Kang S. (2013). Effects of exercise and diet change on cognition function and synaptic plasticity in high fat diet induced obese rats. *Lipids Health Dis*.

[16] Friedwald WT., Levy RI., Fredrickson DS. (1972). Estimation of the concentration of low-density lipoprotein cholesterol in plasma, without use of the preparative ultracentrifuge. *Clin Chem*.

[17] Martins AR., Nachbar RT., Gorjao R., Vinolo MA., Festuccia WT., Lambertucci RH., Cury-Boaventura MF., Silveira LR., Curi R., Hirabara SM. (2012). Mechanisms underlying skeletal muscle insulin resistance induced by fatty acids: importance of the mitochondrial function. *Lipids Health Dis*.

[18] Rivas DA., Yaspelkis BB., Hawley JA., Lessard SJ. (2009). Lipid-induced mTOR activation in rat skeletal muscle reversed by exercise and 5’-aminoimidazole-4-carboxamide-1-beta-D-ribofuranoside. *J Endocrinol*.

[19] Farias JM., Maggi RM., Tromm CB., Silva LA., Luciano TF., Marques SO., Lira FS., de Souza CT., Pinho RA. (2012). Exercise training performed simultaneously to a high-fat diet reduces the degree of insulin resistance and improves adipoR1-2/APPL1 protein levels in mice. *Lipids Health Dis*.

[20] Kang S., Kim KB., Shin KO. (2013). Exercise training improves leptin sensitivity in peripheral tissue of obese rats. *Biochem Biophys Res Commun*.

[21] Thoreen CC., Kang S., Chang J., Liu Q., Zhang J., Gao Y., Reichling LJ., Sim T., Sabatini DM., Gray NS. (2009). An ATP-competitive mammalian target of rapamycin inhibitor reveals rapamycin-resistant functions of mTORC1. *J Biol Chem*.

[22] Kumar A., Harris TE., Keller SR., Choi KM., Magnuson MA., Lawrence JC. (2008). Muscle-specific deletion of rictor impairs insulin-stimulated glucose transport and enhances basal glucogen synthase activity. *Mol Cell Biol*.

[23] Lang CH. (2006). Elevated plasma free fatty acids decrease basal protein synthesis, but not the anabolic effect of leucine, in skeletal muscle. *Am J Physiol Endocrinol Metab*.

[24] Wojtaszewski JF., Richter EA. (2006). Effects of acute exercise and training on insulin action and sensitivity: focus on molecular mechanisms in muscle. *Essays Biochem*.

[25] Lee SS., Kang S. (2015). Effects of regular exercise on obesity and type 2 diabete mellitus in Korean children: improvements glycemic control and serum adipokines level. *J Phys Ther Sci*.

[26] Hornberger TA., Sukhija KB., Chien S. (2006). Regulation of mTOR by mechanically induced signaling events in skeletal muscle. *Cell Cycle*.

[27] Toschi A., Lee E., Xu L., Garcia A., Gadir N., Foster DA. (2009). Regulation of mTORC1 and mTORC2 complex assembly by phosphatidic acid: competition with rapamycin. *Mol Cell Biol*.

[28] Bolster DR., Kubica N., Crozier SJ., Williamson DL., Farrell PA., Kimball SR., Jefferson LS. (2003). Immediate response of mammalian target of rapamycin (mTOR)-mediated signaling following acute resistance exercise in rat skeletal muscle. *J Physiol*.

[29] Mascher H., Andersson H., Nilsson PA., Ekblom B., Blomstrand E. (2007). Changes in signaling pathways regulating protein systhesis in human muscle in the recovery period after endurance exercise.. *Acta Physiol (Oxf)*.

[30] Mascher H., Tannerstedt J., Brink-Elfegoun T., Ekblom B., Gustafsson T., Blomstrand E. (2008). Repeated resistance exercise training induces different changes in mRNA expression of MAFbx and MuRF-1 in human skeletal muscle. *Am J Physiol Endocrinol Metab*.

[31] Kirwan JP., Del Aguila LF., Hernandez JM., Williamson DL., O’Gorman DJ., Lewis R., Krishnan RK. (2000). Regular exercise enhances insulin activation of IRS-1-associated PI3-kinase in human skeletal muscle. *J Appl Physiol*.

[32] Chibalin AV., Yu M., Ryder JW., Song XM., Galuska D., Krook A., Wallberg-Henriksson H., Zierath JR. (2000). Exercise-induced changes in expression and activity of proteins involved in insulin signal transduction in skeletal muscle: differential effects on insulin-receptor substrates 1 and 2. *Proc Natl Acad Sci U S A*.

[33] Liao B., Xu Y. (2011). Exercise improves skeletal muscle insulin resistance without reduced basal mTOR/S6K1 signaling in rats fed a high-fat diet. *Eur J Appl Physiol*.

[34] Meehan CA., Cochran E., Mattingly M., Gorden P., Brown RJ. (2015). Mild Caloric Restriction Decreases Insulin Requirements in Patients With Type 2 Diabetes and Severe Insulin Resistance. *Medicine (Baltimore)*.

[35] Lee HO., Yim JE., Kim YS., Choue R. (2014). Moderate diet-induced weight loss is associated with improved insulin sensitivity in middle-aged healthy obese Korean women. *Nutr Res Pract*.

[36] Larson-Meyer DE., Heilbronn LK., Redman LM., Newcomer BR., Frisard MI., Anton S., Smith SR., Alfonso A., Ravussin E. (2006). Effect of calorie restriction with or without exercise on insulin sensitivity, beta-cell function, fat cell size, and ectopic lipid in overweight subjects. *Diabetes Care*.

[37] Ross R., Dagnone D., Jones PJ., Smith H., Paddags A., Hudson R., Janssen I. (2000). Reduction in obesity and related comorbid conditions after diet-induced weight loss or exercise-induced weight loss in men. A randomized, controlled trial. *Ann Intern Med*.

[38] Fontana L., Villareal DT., Weiss EP., Racette SB., Steger-May K., Klein S., Holloszy JO. (2007). Calorie restriction or exercise: effects on coronary heart disease risk factors. A randomized, controlled trial. *Am J Physiol Endocrinol Metab*.

